# Influence of intraoperative blood salvage and autotransfusion on tumor recurrence after deceased donor liver transplantation: a large nationwide cohort study

**DOI:** 10.1097/JS9.0000000000001683

**Published:** 2024-06-07

**Authors:** Mengfan Yang, Xuyong Wei, Wenzhi Shu, Xiangyu Zhai, Zhisheng Zhou, Jinzhen Cai, Jiayin Yang, Bin Jin, Shusen Zheng, Xiao Xu

**Affiliations:** a Department of Organ Transplantation, Qilu Hospital of Shandong University; b Department of Hepatobiliary Surgery, The Second Hospital, Shandong University, Jinan; c Department of Hepatobiliary and Pancreatic Surgery, Affiliated Hangzhou First People’s Hospital; d Zhejiang University School of Medicine; e Department of Hepatobiliary and Pancreatic Surgery, Shulan (Hangzhou) Hospital; f National Center for Healthcare Quality Management in Liver Transplant; g Key Laboratory of Integrated Oncology and Intelligent Medicine of Zhejiang Province, Hangzhou; h Department of Liver Surgery and Liver Transplantation Center, West China Hospital of Sichuan University, Chengdu; i Organ Transplantation Center, Affiliated Hospital of Qingdao University, Qingdao, People’s Republic of China

**Keywords:** cumulative recurrence rate, deceased donor liver transplantation, hepatocellular carcinoma, intraoperative blood salvage and autotransfusion, Milan criteria, α-Fetoprotein

## Abstract

**Background and aims:**

The practice of intraoperative blood salvage and autotransfusion (IBSA) during deceased donor liver transplantation for hepatocellular carcinoma (HCC) can potentially reduce the need for allogeneic blood transfusion. However, implementing IBSA remains debatable due to concerns about its possible detrimental effects on oncologic recurrence.

**Methods:**

This study retrospectively enrolled nationwide recipients of deceased donor liver transplantation for HCC between 2015 and 2020. The focus was on comparing the cumulative recurrence rate and the recurrence-free survival rate. Propensity score matching was conducted repeatedly for further subgroup comparison. Recipients were categorized based on the Milan criteria, macrovascular invasion, and pretransplant α-Fetoprotein (AFP) level to identify subgroups at risk of HCC recurrence.

**Results:**

A total of 6196 and 329 patients were enrolled in the non-IBSA and IBSA groups in this study. Multivariable competing risk regression analysis identified IBSA as independent risk factors for HCC recurrence (*P*<0.05). Postmatching, the cumulative recurrence rate and recurrence-free survival rate revealed no significant difference in the IBSA group and non-IBSA group (22.4 vs. 16.5%, *P*=0.12; 60.3 vs. 60.9%, *P*=0.74). Recipients beyond Milan criteria had higher, albeit not significant, risk of HCC recurrence if receiving IBSA (33.4 vs. 22.5%, *P*=0.14). For recipients with macrovascular invasion, the risk of HCC recurrence has no significant difference between the two groups (32.2 vs. 21.3%, *P*=0.231). For recipients with an AFP level <20 ng/ml, the risk of HCC recurrence was comparable in the IBSA group and the non-IBSA group (12.8 vs. 18.7%, *P*=0.99). Recipients with an AFP level ≥20 ng/ml, the risk of HCC recurrence was significantly higher in the IBSA group. For those with an AFP level ≥400 ng/ml, the impact of IBSA on the cumulative recurrence rate was even more pronounced (49.8 vs. 21.9%, *P*=0.011).

**Conclusions:**

IBSA does not appear to be associated with worse outcomes for recipients with HCC exceeding the Milan criteria or with macrovascular invasion. IBSA could be confidently applied for recipients with a pretransplant AFP level <20 ng/ml. For recipients with AFP levels ≥20 ng/ml, undertaking IBSA would increase the risk of HCC recurrence.

## Introduction

Highlights

- Intraoperative blood salvage and autotransfusion (IBSA) does not appear to be associated with worse outcomes for recipients exceeding the Milan criteria or with vascular invasion.
- IBSA could be confidently applied for recipients with a pretransplant α-Fetoprotein (AFP) level <20 ng/ml.
- For recipients with AFP levels ≥20 ng/ml, undertaking IBSA would increase the risk of hepatocellular carcinoma recurrence.

Liver transplantation (LT) is widely recognized as an effective treatment for hepatocellular carcinoma (HCC). LT can radically remove tumor lesions while simultaneously addressing primary liver disease^1^. Data from the China Liver Transplant Registry (CLTR) revealed that, on average, ~6000 LT cases were recorded annually between 2018 and 2020 in China. Of these, 35.0% were HCC LT cases. Over the past two decades, there has been a significant reduction in blood transfusion volume during LT, thanks to the continual refinement of surgical techniques, and cases of transfusion-free LT have been reported^2,3^. Despite these advancements, many cases often experience large-volume blood loss. This is typically due to impaired coagulation, an abundance of collateral vessels resulting from portal hypertension, and the surgical processes involved in the anastomosis of the major vessels. Consequently, large-volume blood and plasma transfusions are frequently required^4–6^.

Allogeneic blood transfusion, while vital, has been associated with numerous drawbacks. These include heightened postoperative complications and mortality, extended hospital stays and associated costs, and increased HCC recurrence^7–9^. Research has shown that allogeneic transfusion-related immune modulation can diminish the functioning of T-lymphocytes and natural killer cells, increase T-suppressor cells, and reduce the quantity of macrophages and monocytes^10^. As a response to these challenges, intraoperative blood salvage and autotransfusion (IBSA), a process that recovers shed blood from the surgical site, was introduced in 1943 to lessen the need for allogeneic blood transfusion^11^. Autologous blood transfusion is linked to the activation of natural killer cells and heightened cytokine production, thus enhancing immunocompetence^12^. IBSA has been widely accepted during major abdominal surgery and LT for nonmalignant diseases. However, concerns have been raised about the potential risk of HCC recurrence when using autologous blood in HCC LT. This concern arises from the hypothesis that this method could spread malignant cells, a topic that remains contentious^13^.

While IBSA inevitably results in the reinfusion of circulating tumor cells (CTCs) into the bloodstream, potentially causing metastasis, factors such as the quantity, viability, and metastatic potential of CTCs should be considered. Evidence suggests that the amounts of CTCs found in salvaged and single-filtered leukoreduced autologous blood are higher in patients with HCC beyond the Milan criteria compared to those within the Milan criteria^14^. Studies have shown that α-Fetoprotein (AFP) and macrovascular invasion could independently predict the recurrence-free survival rate for both LT and surgical resection of HCC^15–17^. The major nodule diameter and total tumor count should also be considered^17,18^. Based on these findings, patients undergoing deceased donor LT (DDLT) for HCC should be categorized to ascertain the risk of HCC recurrence after IBSA. Hence, a nationwide multicenter study was conducted to investigate further the association between IBSA and post-transplant HCC recurrence, including a stratified subgroup analysis.

## Methods

### Subjects

A retrospective review was conducted on the prospectively maintained LT database of the CLTR. The initial data collection included patients who underwent adult-to-adult DDLT for HCC in China between January 2015 and December 2020. Exclusion criteria included: patients under 18 years of age, living donor LT, presence of extrahepatic metastasis, combined kidney transplantation, reduced-size or split LT, retransplantation, and missing data on analyzed variables. The remaining recipients were divided into IBSA and non-IBSA groups, depending on whether they received salvaged blood autotransfusion during the LT.

The primary outcome was the overall incidence of HCC recurrence (intrahepatic or extrahepatic) after LT, defined as the survival time leading up to HCC recurrence. Secondary outcomes included the duration until recipient mortality.

All liver grafts were obtained from civilian organ donations, with no grafts sourced from executed prisoners. Each LT was approved by the respective institution’s Liver Transplantation Committee. The work has been reported in line with the strengthening the reporting of cohort, cross-sectional, and case–control studies in surgery (STROCSS) criteria^19^ (Supplemental Digital Content 1, http://links.lww.com/JS9/C724). This study was approved by the CLTR, and the Ethics Committee, in accordance with the ethical principles of the Declaration of Helsinki.

### Data source

The data extracted included relevant donor, recipient, and outcome details such as age, sex, BMI (kg/m^2^), Model for End-Stage Liver Disease (MELD) score, Child-Pugh score, quantity of allogeneic blood transfusion, use of IBSA, intraoperative blood loss, primary liver disease, preoperative AFP level, tumor number, tumor size, Edmondson–Steiner grade, macrovascular invasion, warm ischemia time (WIT), and cold ischemia time (CIT). The outcomes were defined in terms of time until HCC recurrence and time until the recipient’s mortality. The diagnosis of tumor recurrence was based on computerized tomography (CT) scans and serum AFP levels.

The Milan criteria (whether within or beyond) were based on factors including the number and size of tumors. All multicentric HCC nodules and intrahepatic metastasis occurrences were counted when defining the Milan criteria, but completely necrotic nodules were not included.

### Blood salvage policy

A Cell Saver 5 (Haemonetics, Braintree) or Cell Saver 3000P (Jingjing) autotransfusion device was utilized for routine intraoperative blood salvage. Surgically shed blood was suctioned from the surgical field into a reservoir, accompanied by premeasured anticoagulants. Once enough shed blood was amassed, red blood cell (RBC) concentrates were automatically centrifuged, washed, and recollected in reinfusion bags. The 500 ml of blood collected was filtered using a leukocyte depletion filter, followed by autotransfusion, aiming to achieve a target intraoperative blood hemoglobin level of 80 g/l. The blood salvage process commenced after the ascites had been removed and persisted until the completion of the vascular anastomoses.

### Surgical management

All recipients in this study underwent orthotopic LT or piggyback LT based on hemodynamic stability. Intravenous methylprednisolone (10 mg/kg) and basiliximab (20 mg) were administered during the operation as immunosuppressive induction. The recipients were treated with a quadruple regimen of tacrolimus, mycophenolate mofetil, basiliximab, and methylprednisolone. All hepatitis B virus (HBV) infected patients were treated with entecavir or tenofovir combined with hepatitis B immunoglobulin to maintain a high concentration of HBsAb since the operation.

### Statistical analysis

Data normality was checked with Kolmogorov–Smirnov or Shapiro–Wilk test. Student’s *t*-test was used to examine normally scattered continuous data, represented as mean±SD. Median and interquartile ranges were calculated for continuous data with no normal distribution, and the Mann–Whitney *U* test was used for statistical significance. Fisher’s exact test or Pearson’s *χ* ^2^ test was used to examine categorical variables. To analyze the recurrence of HCC, a competing risk regression model (Fine and Gray model) was employed. Multivariable analysis was conducted using a stepwise backward selection method, with *P*<0.10 as the threshold for variable inclusion and *P*>0.10 for variable removal. The Kaplan—Meier survival analysis evaluated the recurrence-free survival rate, with differences in curves tested using the Log-rank test. Propensity score matching (PSM) was implemented using Greedy matching to mitigate the influence of potential confounding factors. This was based on competing risk regression, applying a caliper of 0.2 SD of the log of the propensity score. The balance postmatching was diagnosed using absolute standardized mean differences, all of which were <10% after matching. The result of standardized mean differences is equal to the difference between the mean values of two groups divided by the SD after merging the two groups of data. Statistical analyses were conducted using SAS software (version 9.1) and R software (version 3.6.1), with *P*<0.05 deemed statistically significant.

## Results

### Demographic and clinical parameters of the study population

This study included 329 recipients who received intraoperative blood salvage autotransfusion (IBSA group) and 6196 recipients who did not receive IBSA (non-IBSA group) during LT for HCC. Supplementary Table 1 (Supplemental Digital Content 2, http://links.lww.com/JS9/C725) presents the baseline clinical characteristics of these recipients. The average follow-up period was 24.2 months (ranging from 12.4 to 37.4 months). Before matching, the two groups displayed significant heterogeneity, as indicated by a *P*-value <0.05 and standardized mean differences >10% across various variables.

The average donor age and graft weight were significantly higher in the IBSA group than in the non-IBSA group (*P*<0.05 for both). The mean age of the recipients in the IBSA group was also greater than in the non-IBSA group (*P*<0.001). In both groups, the most common cause of primary liver disease was HBV infection (89.3% in the IBSA group vs. 87.5% in the non-IBSA group, *P*<0.05). Both the Child-Pugh score and the MELD score were higher in the IBSA group than in the non-IBSA group (*P*<0.05), indicating a relatively severe preoperative condition of end-stage liver disease in these patients. The duration was comparable between the two groups [18 (5,41) in the IBSA group vs 17 (4,37) in the non-IBSA group, *P*=0.973].

There were no significant differences in serum AFP levels between the groups. The size of the largest viable tumor was more significant in the non-IBSA group (*P*<0.001), while the number of viable tumors was similar in both groups. The CIT was longer in the IBSA group, and the WIT was nearly the same in both groups. Both intraoperative blood loss and allogeneic blood transfusion were higher in the IBSA group than in the non-IBSA group (*P*<0.001 for both). The median volume of blood autotransfused in the IBSA group was 500 ml (ranging from 400 to 1000 ml).

### Recurrence outcomes for the entire cohort

Cumulative and recurrence-free survival rates were compared between the two groups (Supplementary Figure 1A and B, Supplemental Digital Content 3, http://links.lww.com/JS9/C726). In the IBSA group, the cumulative recurrence rates at 1, 3, and 5 years were 10.7, 19.8, and 22.5%, respectively, while the recurrence-free survival rates at these same intervals were 74.7, 58.9, and 52.2%. In contrast, the non-IBSA group showed cumulative recurrence rates at 1, 3, and 5 years of 9.7, 15.4, and 18.2%, respectively, and recurrence-free survival rates at these intervals were 78.3, 64.0, and 54.9%. Although both the cumulative recurrence rates and recurrence-free survival rates in the IBSA group were lower than those in the non-IBSA group, the difference was not statistically significant [HR=1.199 (0.906–1.586), *P*=0.2 for cumulative recurrence rate; HR=1.182 (0.979–1.426), *P*=0.082 for recurrence-free survival rate].

### Competing risk regression analysis of recurrence outcomes

Both univariable and multivariable competing risk regression analyses (using the Fine and Gray model) were employed to identify risk factors associated with HCC recurrence post-LT in the entire study cohort (Table 1). Univariable competing risk regression analysis indicated that donation type, donor BMI, recipient age and sex, underlying liver disease etiology, Child-Pugh score, MELD score, AFP level, tumor count, largest tumor size, macrovascular invasion, tumor differentiation, graft CIT, intraoperative allogeneic blood transfusion, and intraoperative autologous blood transfusion were all potential risk factors for HCC recurrence (*P*<0.10). Multivariable analysis revealed that recipient MELD score, AFP level, tumor count, largest tumor size, macrovascular invasion, tumor differentiation, graft CIT, intraoperative allogeneic blood transfusion, and intraoperative autologous blood transfusion were independently associated with HCC recurrence (*P*<0.05).

**Table 1:** Multivariable analysis for recurrence-free survival in HCC LT recipients.

	Univariable		Multivariable	
	HR (95% CI)	*P*	HR (95% CI)	*P*
Age (year)	1.001 (0.997–1.005)	0.740		
Sex, male	1.031 (0.889–1.197)	0.690		
Donor type, DCD	0.928 (0.820–0.968)	0.007	0.926 (0.842–1.019)	0.120
BMI (kg/m^2^)	0.982 (0.964–1.000)	0.054	1.049 (0.985–1.118)	0.141
Age (year)	0.985 (0.979–0.991)	0.000	0.995 (0.987–1.003)	0.190
Sex, male	0.752 (0.612–0.923)	0.006	0.844 (0.649–1.097)	0.200
Etiology, HBV	0.908 (0.848–0.971)	0.005	0.941 (0.867–1.022)	0.150
BMI (kg/m^2^)	1.005 (0.998–1.012)	0.140		
MELD	0.992 (0.988–0.997)	<0.001	1.009 (1.000–1.019)	0.041
Child-Pugh	0.928 (0.911–0.945)	<0.001	0.947 (0.862–1.040)	0.102
AFP, >20 ng/ml	1.868 (1.736–2.011)	<0.001	1.491 (1.361–1.634)	<0.001
Number of tumors, solitary	1.315 (1.123–1.540)	<0.001	1.364 (1.184–1.570)	<0.001
Size of the largest tumor, ≥5 cm	2.663 (2.370–2.992)	<0.001	2.063 (1.773–2.401)	<0.001
Microvascular invasion, yes	2.826 (2.525–3.163)	<0.001	1.710 (1.462–1.999)	<0.001
Tumor differentiation	2.226 (1.939–2.554)	<0.001	1.429 (1.175–1.739)	<0.001
WIT (min)	0.997 (0.989–1.005)	0.450		
CIT (h)	1.063 (1.043–1.083)	<0.001	1.052 (1.024–1.081)	<0.001
Intraoperative blood loss (100 ml)	1.005 (0.998–1.012)	0.140		
Intraoperative allogeneic blood transfusion (100 ml)	1.008 (0.997–1.019)	0.064	1.212 (1.011–1.453)	0.029
Intraoperative autologous blood transfusion (100 ml)	1.010 (0.998–1.022)	0.088	1.347 (1.072–1.691)	0.010

### Recurrence outcomes after propensity score matching

Subsequently, PSM procedure at a ratio of 1:4 was conducted to balance the differences in baseline clinical characteristics recognized as independent risk factors between the two groups. In addition to these factors, the donor age, graft weight, recipient age, and intraoperative blood loss were also considered.

After matching, we selected 1198 recipients from the non-IBSA group and 320 recipients from the IBSA group. Table 2 shows the baseline clinical characteristics of the patients in the two groups. We observed no significant differences in the donor, recipient, and perioperative clinical characteristics between the groups, and all matched variables showed standardized mean differences of less than 10%. All risk factors—including MELD score, pretransplant treatment, AFP level, tumor count, largest tumor size, macrovascular invasion, tumor differentiation, graft CIT, intraoperative allogeneic blood transfusion, and intraoperative autologous blood transfusion—were adjusted.

**Table 2:** Clinical characteristics of nonautotransfusion group and autotransfusion group after matching.

	Non-IBSA (*n*=1198)	IBSA (*n*=320)	*P*	SMD
Donor				
Age (year)	46.4±13.0	46.5±12.6	0.884	0.009
Sex, male	1006 (84.0%)	271 (84.7%)	0.756	0.008
Donor type, DCD	575 (48.0%)	157 (49.1%)	0.735	0.022
BMI (kg/m^2^)	23.1±3.1	23.2±3.3	0.688	0.028
Graft weight (g)	1399.8±260.5	1414.3±341.2	0.566	0.051
Recipient				
Age (year)	54.4±8.9	54.6±8.6	0.865	0.011
Sex, male	1066 (89.0%)	280 (87.5%)	0.458	0.017
Etiology of cirrhosis			0.768	0.020
Hepatitis B	1039 (86.7%)	272 (85.0%)		
Hepatitis C	24 (2.0%)	5 (1.6%)		
Alcoholic	54 (4.5%)	15 (4.7%)		
Autoimmune	7 (0.6%)	2 (0.6%)		
Other	74 (6.2%)	26 (8.1%)		
BMI (kg/m^2^)	23.9±3.1	23.8±3.3	0.656	0.030
MELD	24.8±12.7	24.3±12.3	0.543	0.038
Child-Pugh			0.497	0.042
A	193 (16.1%)	44 (13.8%)		
B	372 (31.1%)	97 (30.3%)		
C	633 (52.8%)	179 (55.9%)		
Waiting time (d)	17 (4, 38)	17 (4, 37)	0.894	0.018
Pretransplant TACE	346 (28.9%)	85 (26.6%)	0.414	0.082
Pretransplant RFA	175 (14.6%)	43 (13.4%)	0.596	0.081
Serum AFP at transplant (ng/ml)			0.916	0.026
<20	609 (50.8%)	163 (50.9%)		
20–400	338 (28.2%)	93 (29.1%)		
>400	251 (21.0%)	64 (20.0%)		
Number of viable tumors	2.0±2.7	2.0±2.2	0.805	0.016
Size of the largest viable tumor	3.9±3.1	4.0±4.0	0.812	0.017
Microvascular invasion	184 (15.4%)	52 (16.3%)	0.696	0.057
Tumor differentiation			0.817	0.057
Well differentiated	180 (15.0%)	45 (14.1%)		
Moderately differentiated	912 (76.1%)	249 (77.8%)		
Poorly differentiated	106 (8.8%)	26 (8.1%)		
Perioperative				
Operative time (h)	7.2±1.9	7.1±2.7	0.516	0.072
WIT (min)	4.8±6.4	4.9±6.0	0.745	0.021
CIT (h)	7.0±2.7	7.2±2.5	0.151	0.090
Intraoperative blood loss (ml)	1400 (700–2400)	1500 (800–2500)	0.117	0.095
Intraoperative allogeneic blood transfusion (U)	2 (0–6)	2 (0–6)	0.521	0.062
Intraoperative autologous blood transfusion (ml)	–	500 (400–1000)	–	–

**Figure 1:**
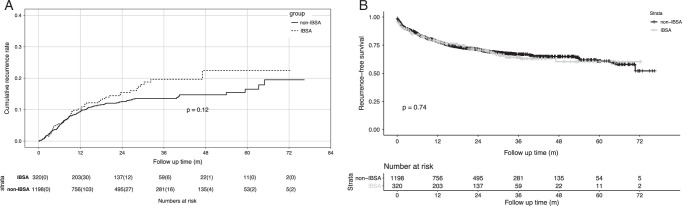
Comparison of cumulative recurrence rates and recurrence-free survival rates after propensity score matching. (A) Cumulative recurrence rate and (B) recurrence-free survival rate. IBSA, intraoperative blood salvage and autotransfusion.

We compared the cumulative and recurrence-free survival rates between the two matched groups (Fig. 1A and B). In the IBSA group, the 1-year, 3-year, and 5-year cumulative recurrence rates were 10.1, 19.7, and 22.4%, respectively, while the 1-year, 3-year, and 5-year recurrence-free survival rates were 78.6, 64.3, and 60.3%, respectively. For the non-IBSA group, the 1-year, 3-year, and 5-year cumulative recurrence rates were 9.4, 13.8, and 16.5%, respectively, and the 1-year, 3-year, and 5-year recurrence-free survival rates were 78.2, 66.9, and 60.9%, respectively. The IBSA group exhibited lower cumulative recurrence rates and recurrence-free survival rates than the non-IBSA group; however, these differences were not statistically significant [HR=1.293 (0.937–1.783), *P*=0.12; HR=1.039 (0.827–1.306), *P*=0.74].

### Subgroup analysis stratified by Milan criteria

**Figure 2:**
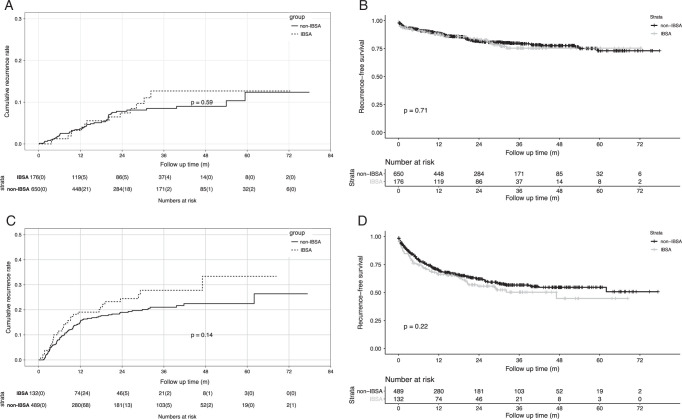
Comparison of cumulative recurrence rates and recurrence-free survival rates stratified by Milan criteria. (A, B) Cumulative recurrence rate (A) and recurrence-free survival rate (B) between IBSA and non-IBSA group within Milan criteria. (C, D) Cumulative recurrence rate (C) and recurrence-free survival rate (D) between IBSA and non-IBSA groups beyond Milan criteria. IBSA, intraoperative blood salvage and autotransfusion.

Subgroup analysis stratified by Milan Criteria was adopted to identify the interval that affected HCC recurrence. PSM was conducted at a ratio of 1:4 for patients within or beyond Mila criteria, the matching factors were as mentioned ahead. As shown in Figure 2A, 176 recipients in IBSA group and 650 recipients in non-IBSA group within Milan criteria were enrolled. HCC recurrence risk was not significantly different between the two groups within Milan criteria (HR=1.179 (0.650–2.140), *P*=0.59), and the 5-year cumulative recurrence rates were 12.4 and 12.7%. Recurrence-free survival rates were also similar between the two groups within Milan criteria [Fig. 2B, HR=1.079 (0.728–1.599), *P*=0.71], and the 5-year recurrence-free survival rates were 73.1 and 75.4% in non-IBSA group and IBSA group, respectively.

For recipients beyond Milan criteria, 132 recipients in IBSA group and 489 recipients in non-IBSA group were enrolled. The risk of HCC recurrence was slightly higher in the IBSA group than in the non-IBSA group (Fig. 2C); the 5-year cumulative recurrence rates were 33.4 and 22.5% [HR=1.354 (0.905–2.024), *P*=0.14], respectively. There was a worse recurrence-free survival rate among IBSA patients than among non-IBSA patients [Fig. 2D, HR=1.202 (0.893–1.618), *P*=0.22], and the 5-year recurrence-free survival rates were 54.7 and 44.7% in IBSA group and non-IBSA group, respectively.

### Subgroup analysis stratified by macrovascular invasion

**Figure 3:**
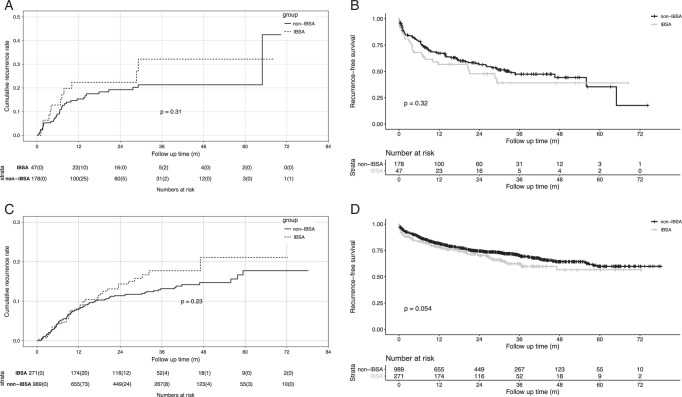
Comparison of cumulative recurrence rates and recurrence-free survival rates stratified by macrovascular invasion. (A, B) Cumulative recurrence rate (A) and recurrence-free survival rate (B) between IBSA and non-IBSA groups with macrovascular invasion. (C, D) Cumulative recurrence rate (C) and recurrence-free survival rate (D) between IBSA and non-IBSA groups without macrovascular invasion. IBSA, intraoperative blood salvage, and autotransfusion.

Furthermore, PSM and subgroup analysis stratified by macrovascular invasion was adopted. As shown in Figure 3A, 47 recipients in IBSA group and 178 recipients in non-IBSA group with macrovascular invasion were enrolled. The risk of HCC recurrence was not significantly different between the two groups with macrovascular invasion [HR=1.402 (0.727–2.706), *P*=0.231], and the 5-year cumulative recurrence rates were 32.2 and 21.3% in IBSA group and non-IBSA group. Recurrence-free survival rates were also similar between the two groups with macrovascular invasion [Fig. 3B, HR=1.258 (0.802–1.973), *P*=0.32], and the 5-year recurrence-free survival rates were 39.1 and 35.4% in IBSA group and non-IBSA group, respectively.

For recipients without macrovascular invasion, 271 recipients in IBSA group and 989 recipients in non-IBSA group were enrolled. The risk of HCC recurrence showed insignificant difference in IBSA group and non-IBSA group (Fig. 3C); the 5-year cumulative recurrence rates were 21.1 and 17.7% [HR=1.251 (0.865–1.809), *P*=0.23], respectively. IBSA patients had worse but not significant recurrence-free survival rates than non-IBSA patients [Fig. 3D, HR=1.270 (0.995–1.622), *P*=0.054], and the 5-year recurrence-free survival rates were 56.6 and 59.8% in IBSA group and non-IBSA group, respectively.

### Subgroup analysis stratified by AFP level

We conducted PSM and subgroup analysis stratified according to the AFP level before LT. The analysis revealed a variance in HCC recurrence risk based on the AFP levels and the specific group.

**Figure 4:**
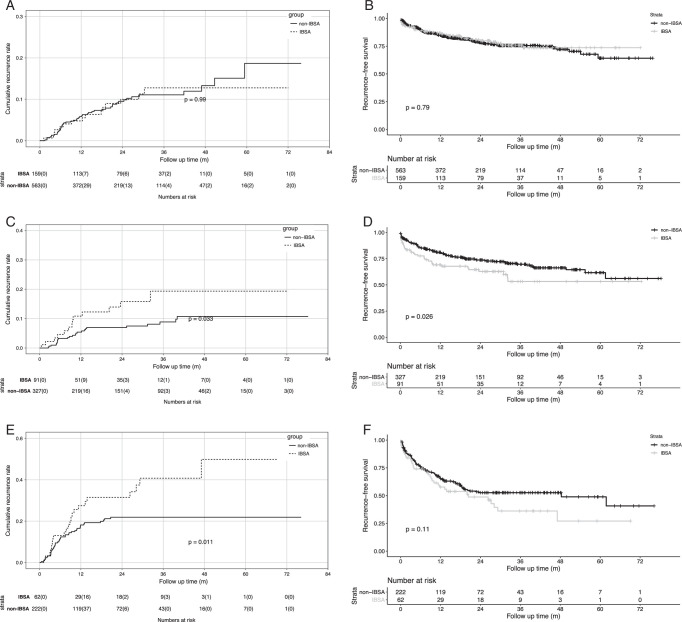
Comparison of cumulative recurrence rates and recurrence-free survival rates stratified by pretransplant AFP levels. (A, B) Cumulative recurrence rate (A) and recurrence-free survival rate (B) between IBSA and non-IBSA group with AFP level <20 ng/ml. (C, D) Cumulative recurrence rate (C) and recurrence-free survival rate (D) between IBSA and non-IBSA group with AFP level 20–400 ng/ml. (E, F) Cumulative recurrence rate (E) and recurrence-free survival rate (F) between IBSA and non-IBSA group with AFP level ≥400 ng/ml. AFP, α-Fetoprotein; IBSA, intraoperative blood salvage and autotransfusion.

In the subgroup with AFP levels lower than 20 ng/ml, 159 recipients in IBSA group and 563 recipients in non-IBSA group were enrolled. There was no notable disparity in the risk of HCC recurrence between the IBSA and the non-IBSA groups [HR=1.005 (0.565–1.785), *P*=0.99, as shown in Fig. 4A]. The 5-year cumulative recurrence rates were 12.8% for the IBSA group and 18.7% for the non-IBSA group. Similarities were also observed in the recurrence-free survival rates for the two groups [HR=0.949 (0.641–1.405), *P*=0.79, as displayed in Fig. 4B], with rates standing at 73.6% for the IBSA group and 64.2% for the non-IBSA group after a span of 5 years.

In contrast, for the recipients with AFP levels of 20–400 ng/ml, 91 recipients in IBSA group and 327 recipients in non-IBSA group enrolled, the IBSA group exhibited a significantly elevated risk of HCC recurrence compared to the non-IBSA group [HR=2.060 (1.059–4.005), *P*=0.033, as delineated in Fig. 4C]. The 5-year cumulative recurrence rates for this group were 19.4% for the IBSA group and 10.7% for the non-IBSA group. The recurrence-free survival rates for the IBSA group were significantly lower than the non-IBSA group [HR=1.561 (1.051–2.316), *P*=0.026, as presented in Fig. 4D], with 5-year rates being 53.2 and 61.7% for the IBSA and the non-IBSA groups, respectively.

Specifically, for recipients with AFP levels of 400 ng/ml or more, 62 recipients in IBSA group and 222 recipients in non-IBSA group enrolled, the risk of HCC recurrence remained significantly higher in the IBSA group relative to the non-IBSA group [HR=1.909 (1.157–3.148), *P*=0.011, as indicated in Fig. 4E]. The 5-year cumulative recurrence rates in this category were 49.8% for the IBSA group and 21.9% for the non-IBSA group. Figure 4F depicts that the recurrence-free survival rates were significantly inferior in the IBSA group compared to the non-IBSA group [HR=1.372 (0.927–2.032), *P*=0.114], with 5-year rates of 27.1% for the IBSA group and 40.7% for the non-IBSA group.

## Discussion

Prior research on surgery for HCC patients has suggested that IBSA can be utilized safely without heightening the risk of HCC recurrence^20,21^. However, most of these studies predominantly focused on living donor LT, which has stricter selection criteria, making these results challenging to apply to DDLT^22^. Additionally, stratified analyses are necessary to identify subgroups with recurrence-free benefits or risks. In this context, a nationwide multicenter study was conducted across 31 provinces in China to investigate the association between IBSA and HCC recurrence after DDLT. The study emphasizes that IBSA should be regarded as risk factor for HCC recurrence in DDLT. Recipients with HCC beyond the Milan criteria or with macrovascular invasion should cautiously treated IBSA during DDLT. Furthermore, for recipients with a pretransplant AFP level ≥20 ng/ml, IBSA should be considered an independent risk factor that could potentially increase the risk of HCC recurrence and reduce the recurrence-free survival rate.

Blood transfusion is sometimes deemed necessary during LT, and allogeneic blood transfusion was primarily employed in traditional practices decades ago^23,24^. Allogeneic blood transfusion has been reported to be associated with infections, hemolytic reactions, allergic reactions, acute lung injury, graft versus host disease, and circulatory failure, and it may even impair the immune function of organ transplant recipients, thereby increasing the risk of HCC recurrence^25–28^. Meta-analysis by Hu *et al*. and Xun *et al*. proved that receipt of perioperative allogeneic blood transfusion for HCC patients undergoing radical hepatectomy is associated with a decrease in overall survival and recurrence-free survival and an increase in complications^29,30^, suggesting that surgeons should avoid perioperative allogenic blood transfusion during hepatectomy. To mitigate the large amount of intraoperative blood loss, IBSA was introduced as a safe and effective method during surgery. Nevertheless, the use of IBSA during oncologic surgery remains controversial.

In the 1990s, Hansen *et al*. strongly supported the contraindication of intraoperative autotransfusion in tumor surgery. They detected tumor cells in the bloodshed during oncologic surgery in 57 of 61 patients, suggesting potential risks of local tumor recurrence or hematogenic metastasis after retransfusion^31^. Subsequently, the IBSA process was refined with the introduction of Cell Saver and leukocyte depletion filters, which could, to some extent, remove tumor cells, thus reducing the risk of tumor recurrence during blood salvage^32^. However, the effectiveness of leukocyte depletion filters remains to be substantiated by high-level clinical research. Liang *et al*. found that HCC cells contaminated the shed blood samples of 20 of 32 LT recipients, and 15 of these samples remained positive even after Cell Saver processing. Furthermore, they noted that recipients within the Milan criteria were less likely to have HCC cell contamination, since such cells were more likely to be removed by the Cell Saver^14^. Gwak *et al*.^32^ argued that leukocyte depletion filters could not fully remove all tumor cells when the HCC cell load in salvaged blood is high.

A substantial body of research suggests that a higher tumor burden (number and size of lesions) and elevated serum AFP levels are correlated with a poor prognosis in HCC patients^33–35^. Furthermore, both AFP levels and maximum tumor size have shown a positive relationship with circulating tumor DNA and CTCs^36,37^. The cut-off value AFP <20 ng/ml is commonly used diagnostic threshold in clinical practice, <20 is considered AFP-negative. Most studies regarding sensitivity and specificity are 46–59% and 87–93% using the cut-off of 20 ng/ml, respectively^38,39^. Pretreatment AFP levels >400 ng/ml were shown to be associated with significantly longer median progression-free survival and overall survival in therapy response^39^. Besides, the Hangzhou criteria for LT includes the serum AFP levels using the cut-off of 400 ng/ml, which not only expands the population for transplantation, also achieves the recurrence-free survival and overall survival rate that is basically consistent with the Milan criteria^40,41^. Setting cutoff values at 20 and 400 can also ensure that each subgroup includes sufficient cases for comparison. It is critical to stratify and analyze subgroups to determine which recipient group can benefit the most from IBSA.

However, numerous retrospective studies have suggested that using a leukocyte depletion filter in the IBSA process during LT for HCC does not increase the risk of HCC recurrence. This indicates that either the number of tumor cells entering the filtration process is limited, the re-entering tumor cells lack the potential for seeding and metastasis, or the autotransfusion process does not alter the natural biological course of the disease^42–44^. A study published in 2005, although potentially biased due to inconsistency in participant selection, compared 16 LT for HCC recipients without IBSA and 31 recipients with IBSA, finding no significant change in the risk of neoplastic recurrence resulting from IBSA^45^. The most extensive retrospective study on living donor LT found no differences in the cumulative HCC recurrence rate at 1, 2, and 5 years among 397 matched recipients, revealing that the IBSA and non-IBSA groups had equivalent risks for overall, intrahepatic, or extrahepatic recurrence^46^. Furthermore, a study of living donor LT recipients with advanced HCC showed no negative impact of IBSA after single leukocyte depletion filters on post-transplant HCC recurrence and survival, with statistically insignificant probabilities favoring the technique^47^. Ivanics *et al*.^48^ reported that IBSA does not appear to adversely affect oncologic outcomes in LT recipients with incidental HCC, as the risk of recurrence in both IBSA and non-IBSA groups was low and comparable.

Our findings indicated no significant differences in cumulative and recurrence-free survival rates between the IBSA and non-IBSA groups, regardless of whether the cohort was matched. However, in contrast to prior studies, our multivariable competing risk regression analysis identified IBSA as an independent risk factor for HCC recurrence. This discrepancy could be because the recipients in our study had advanced tumor biology, with 3246 (49.7%) recipients exceeding the Milan criteria. This comparatively higher tumor burden might explain our differing results. PSM was conducted every time before subgroup analysis to eliminate bias, and the matching factors are the same as before. Subgroup analysis suggested that recipients with HCC beyond the Milan criteria or with macrovascular invasion had increased, although statistically insignificant, the risks of HCC recurrence. In contrast, recipients within the Milan criteria or without macrovascular invasion had similar cumulative recurrence rates and recurrence-free survival rates. For recipients with an AFP level ≥20 ng/ml, we found significantly increased risks of HCC recurrence and lower recurrence-free survival rates. For this group of recipients, IBSA should be viewed as a contraindication.

Our study is not without limitations. Firstly, due to the retrospective and nonrandomized design, we cannot exclude the potential for selection and misclassification bias. As a registry-recorded multicenter study, the follow-up times varied, and some recipients were excluded due to missing clinical data. Inaccurate or incomplete data may lead to biased or misleading research results. Secondly, the tumor characteristics in our study differ from those of East Asian transplant centers due to advanced tumor biology and lower expected post-LT survival probability. Many recipients did not survive due to intraoperative and postoperative complications. Thirdly, as a nationwide multicenter study, the follow-up times varied, and some recipients were excluded due to missing clinical data. Some new crucial diagnostic biomarker as protein induced by vitamin K absence or antagonist-II, was not examined routinely in the early stage. Nonetheless, to our knowledge, our study represents the largest cohort of recipients receiving IBSA during DDLT to date for this retrospective study, and PSM was employed repeatedly before subgroup analysis with the standardized mean differences controlled below 0.1 to mitigate selection bias. Future multicenter randomized controlled trials will be needed to validate these results.

In conclusion, IBSA does not appear to be associated with worse outcomes for recipients with HCC exceeding the Milan criteria or with macrovascular invasion. For recipients with a pretransplant AFP level of ≥20 ng/ml, IBSA should be considered an independent risk factor that could potentially increase the risk of HCC recurrence. For recipients with an AFP level ≥20 ng/ml, further researches are necessary to explore how the HCC recurrence rates increased.

## Ethical approval

Liver transplantation was approved by the liver transplantation committee of each institution and was performed after informed consent was obtained from the patients. The study was approved by the China Liver Transplant Registry with approval number 20210021, and by the ethics committee. It was performed following the ethical principles of the Declaration of Helsinki.

## Consent

Informed consent was waived as previously collected data that did not include personally identifiable information were used.

## Source of funding

This research was partially supported by Youth Program of Natural Science Foundation of Shandong (No. ZR2023QH315); National Natural Science Funds for Distinguished Young Scholar of China (No. 81625003); Key Program, National Natural Science Foundation of China (No. 81930016).

## Author contribution

Our manuscript has 10 authors, all of whom contributed significantly to this study. Conceived and designed the experiments (M.Y. and X.W.), collected the data (M.Y., X.W., W.S., X.Z., and Z.Z.), analyzed the data (M.Y., X.W., and W.S.), wrote the paper (M.Y. and X.W.), and supervised the paper (J.Y., J.C., B.J., S.Z., and X.X.).

## Conflicts of interest disclosure

The authors have no conflicts of interest to declare that pertain to this study.

## Research registration unique identifying number (UIN)

1. Name of registry: Clinical Trials.
2. Unique identifying number or registration ID: NCT06307158.
3. Hyperlink to your specific registration: https://classic.clinicaltrials.gov/ct2/show/NCT06307158.

## Guarantor

Xiao Xu, Zhejiang University School of Medicine, Hangzhou 310058, China. E-mail: zjxu@zju.edu.cn.

## Data availability statement

The data that support the findings of this study are available on request from the corresponding author. The data are not publicly available due to privacy or ethical restrictions.

## Provenance and peer review

Not commissioned, externally peer-reviewed.

## Supplementary Material
