# Supplementary material for: Influence of intraoperative blood salvage and autotransfusion on tumor recurrence after deceased donor liver transplantation: a large nationwide cohort study
Source: Int J Surg. 2024 Jun 7;110(9):5652–61. doi: 10.1097/JS9.0000000000001683 (PMC11392187; doi:10.1097/JS9.0000000000001683)
Supplement: Supplementary file 2 [file js9-110-5652-s002.docx]

Supplementary Table 1. Clinical Characteristics of Non-autotransfusion Group and Autotransfusion Group Before Matching.

|  | Non-IBSA (n=6196) | IBSA (n=329) | *P* value | SMD |
| --- | --- | --- | --- | --- |
| Donor |  |  |  |  |
| Age (yr) | 44.8±14.0 | 46.6±12.6 | 0.018 | 0.134 |
| Gender, male | 5136 (82.9%) | 278 (86.9%) | 0.450 | 0.027 |
| Donor type, DCD | 2770 (44.7%) | 162 (50.6%) | 0.107 | 0.091 |
| BMI (kg/m^2^) | 23.2±3.3 | 23.2±3.3 | 0.921 | 0.070 |
| Graft weight (g) | 1345.0±270.4 | 1413.6±338.1 | 0.008 | 0.248 |
| Recipient |  |  |  |  |
| Age (yr) | 52.3±9.3 | 54.6±8.6 | <0.001 | 0.239 |
| Gender, male | 5555 (89.7%) | 289 (90.3%) | 0.295 | 0.065 |
| Etiology of cirrhosis |  |  | 0.005 | 0.166 |
| Hepatitis B | 5536 (89.3%) | 280 (87.5%) |  |  |
| Hepatitis C | 166 (2.7%) | 5 (1.6%) |  |  |
| Alcoholic | 140 (2.3%) | 15 (4.7%) |  |  |
| Autoimmune | 36 (0.6%) | 2 (0.6%) |  |  |
| Other | 318 (5.1%) | 27 (8.4%) |  |  |
| BMI (kg/m^2^) | 23.8±3.3 | 23.8±3.6 | 0.941 | 0.043 |
| MELD | 19.7±12.2 | 24.5±12.3 | <0.001 | 0.388 |
| Child-Pugh |  |  | <0.001 | 0.312 |
| A | 1275 (20.6%) | 44 (13.4%) |  |  |
| B | 2231 (36.0%) | 100 (30.4%) |  |  |
| C | 2690 (43.4%) | 185 (56.2%) |  |  |
| Waiting time (d) | 18 (5, 41) | 17 (4, 37) | 0.973 | 0.003 |
| Serum AFP at transplant (ng/mL) |  |  | 0.096 | 0.123 |
| <20 | 2872 (44.9%) | 169 (48.1%) |  |  |
| 20-400 | 1801 (31.1%) | 95 (29.0%) |  |  |
| >400 | 1523 (24.0%) | 65 (23.0%) |  |  |
| Number of viable tumors | 2.0±2.3 | 2.0±2.2 | 0.862 | 0.083 |
| Size of the largest viable tumor | 4.9±4.0 | 4.0±4.0 | <0.001 | 0.228 |
| Microvascular invasion | 1358 (21.9%) | 52 (16.3%) | 0.009 | 0.194 |
| Tumor differentiation |  |  | <0.001 | 0.054 |
| Well differentiated | 1338 (21.6%) | 46 (14.0%) |  |  |
| Moderately differentiated | 4176 (67.4%) | 256 (77.8%) |  |  |
| Poorly differentiated | 682 (11.0%) | 27 (8.2%) |  |  |
| Perioperative |  |  |  |  |
| Operative time (h) | 7.2±1.8 | 7.1±2.7 | 0.613 | 0.029 |
| WIT (min) | 5.0±6.3 | 4.9±6.0 | 0.764 | 0.017 |
| CIT (h) | 6.1±2.6 | 7.2±2.6 | <0.001 | 0.422 |
| Intraoperative blood loss (mL) | 1000 (500, 1800) | 1500 (800, 2500) | <0.001 | 0.569 |
| Intraoperative allogeneic blood transfusion (U) | 2 (0, 4) | 2 (0, 6) | 0.010 | 0.286 |
| Intraoperative autologous blood transfusion (mL) | - | 500 (400, 1000) | - | - |
