# Supplementary material for: Influence of intraoperative blood salvage and autotransfusion on tumor recurrence after deceased donor liver transplantation: a large nationwide cohort study
Source: Int J Surg. 2024 Jun 7;110(9):5652–61. doi: 10.1097/JS9.0000000000001683 (PMC11392187; doi:10.1097/JS9.0000000000001683)
Supplement: Supplementary file 3 [file js9-110-5652-s003.pdf]

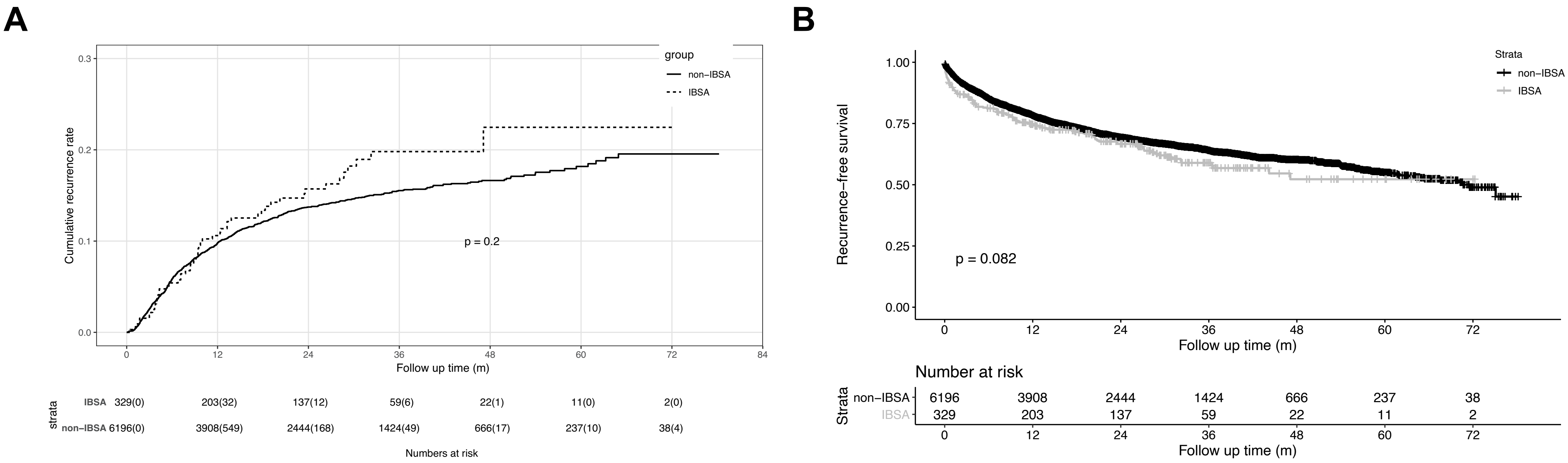

Supplementary Figure 1. Comparison of cumulative recurrence rates and recurrence-free survival rates in the IBSA and non-IBSA groups of the entire cohort. (A) Cumulative recurrence rate and (B) recurrence-free survival rate. IBSA, intraoperative blood salvage and autotransfusion.
